# P-1668. Perioperative Antibiotic Use for Heart Transplantation at Children’s Hospitals in the United States

**DOI:** 10.1093/ofid/ofae631.1834

**Published:** 2025-01-29

**Authors:** Kengo Inagaki, Alison C Tribble

**Affiliations:** University of Michigan, Ann Arbor, Michigan; C.S. Mott Children's Hospital, University of Michigan Health, Ann Arbor, MI

## Abstract

**Background:**

Data on perioperative antibiotic use for pediatric heart transplantation are scarce, although such information can inform the optimal approaches for perioperative antibiotic prophylaxis.

**Figure:**
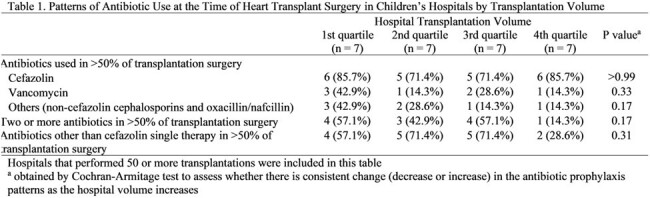

**Methods:**

We identified children undergoing heart transplantation in the Pediatric Health Information System database. The primary outcomes of interest were perioperative antibiotics other than cefazolin single therapy and prolonged duration (defined as more than 1 postoperative calendar days) of postoperative antibiotics.

**Figure 1.**
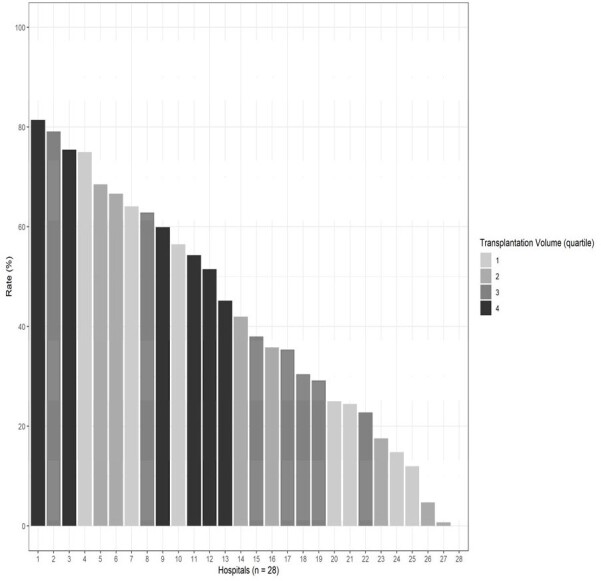
Rate of the Use of Cefazolin Single Therapy at the Time of Transplantation Surgery in Children’s Hospitals by Transplantation Volume Hospitals that performed 50 or more transplantations were included in this Figure The hospital with the rate of 0% (#28) was a 4th-quartile transplantation volume hospital

**Results:**

Cefazolin was used predominantly (i.e. > 50% of cases) as part of the perioperative antibiotic regimen at 22 of 28 (78.6%) hospitals, while only 12 of 28 hospitals (42.9%) predominantly used cefazolin as a single therapy. The predominant use of two or more antibiotics (with or without cefazolin) perioperatively was seen in 12 of 28 hospitals (42.9%). The median postoperative duration of antibiotics was 2 calendar days (interquartile range: 1-3). Patients undergoing transplantation at higher transplantation volume hospitals were less likely to receive antibiotics other than cefazolin single therapy or to receive prolonged duration (odds ratio, OR: 0.45 [95% confidence interval, CI: 0.38-0.53], and OR: 0.76 [95%CI: 0.63-0.91], respectively, for the 4^th^ quartile hospitals).

**Figure 2.**
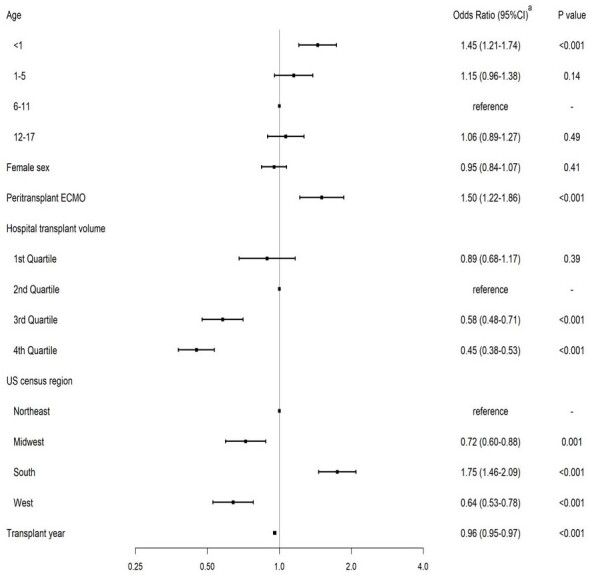
Factors Associated with the Use of Perioperative Antibiotic Other Than Cefazolin Single Therapy among Children Undergoing Heart Transplantation a obtained using multivariable logistic regression Abbreviations: CI, confidence interval; ECMO, extracorporeal membrane oxygenation Hospitals that performed 50 or more transplantations were included

**Conclusion:**

Perioperative antibiotic use for heart transplantation varies substantially across children’s hospitals. Higher transplantation volume hospitals were more likely to use cefazolin single therapy and shorter durations of antibiotics although there remain rooms for improvement. Careful review of the best available evidence should be incorporated in developing institutional perioperative antibiotic approaches for heart transplantation, particularly at low-volume hospitals.

**Figure 3:**
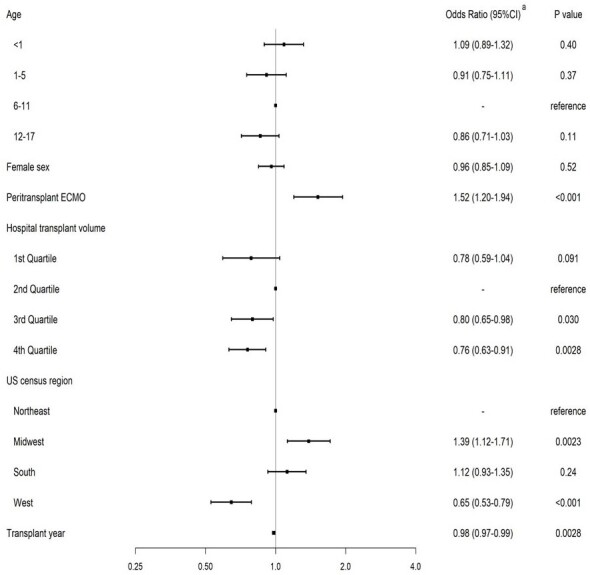
Factors Associated with Postoperative Duration of Antibiotics More Than 1 Calendar Days a obtained using multivariable logistic regression Abbreviations: CI, confidence interval; ECMO, extracorporeal membrane oxygenation Hospitals that performed 50 or more transplantations were included

**Disclosures:**

**Kengo Inagaki, MD**, AstraZeneka: Grant/Research Support

